# Glycolytic Enzymes Are Part of an Oncogenic Network in AML

**DOI:** 10.3390/cells15060569

**Published:** 2026-03-23

**Authors:** Stefan Nagel, Corinna Meyer, Claudia Pommerenke

**Affiliations:** 1 Department of Human and Animal Cell Lines, Leibniz-Institute DSMZ-German Collection of Microorganisms and Cell Cultures, 38124 Braunschweig, Germany; corinna.meyer@dsmz.de; 2 Department of Bioinformatics, Leibniz-Institute DSMZ-German Collection of Microorganisms and Cell Cultures, 38124 Braunschweig, Germany; claudia.pommerenke@dsmz.de

**Keywords:** chromoanagenesis, DYRK1A, glycolysis, KLF3, lactate, NKL-code, NOTCH3, SLC2A1

## Abstract

**Highlights:**

**What are the main findings?**

- Aberrant PFKL overexpression reduces the intra-cellular glucose level in AML cell line OCI-M2.
- The reduced glucose level activates developmental gene activities including IRF6 and its downstream targets.

**What is the implication of the main finding?**

- Cell line OCI-M2 may serve as an in vitro model to establish glycolysis-targeted therapies in AML.

**Abstract:**

Erythroid acute myeloid leukemia (AML) cell line OCI-M2 expresses a particular oncogenic network: IRF6, in concert with ETV2 and HEY1, aberrantly activates NKL homeobox gene NKX2-4, which in turn represses megakaryocytic lineage factor FLI1. Interestingly, in keratinocytes, IRF6 is able to bind glucose which promotes IRF6-dimerization and thus alters its binding site selection. Here, we used OCI-M2 as a model to investigate the role of glucose level and IRF6 in leukemogenesis. Treatment of OCI-M2 with high glucose or 2-deoxy-glucose resulted in the downregulation of IRF6 and NKX2-4, and the upregulation of FLI1, indicating that glucose-mediated dimerization of IRF6 altered its reported autoactivation. The screening of this cell line for genes encoding glycolytic enzymes identified aberrant overexpression of glucose-6-phosphate isomerase (GPI) and phosphofructokinase L (PFKL), which were targeted by genomic amplification and chromothripsis-like alterations, respectively. Furthermore, GPI was activated by NKX2-4 and ETV2, and PFKL by ETV2. Finally, siRNA-mediated downregulation of PFKL resulted in elevated glucose levels, suppressed expression of IRF6 and NKX2-4, and activated FLI1. Thus, we connected an oncogenic regulatory network with deregulated glycolytic enzymes and glucose metabolism, thereby establishing a new in vitro model to develop novel therapeutic avenues in AML subsets.

## 1. Introduction

Acute myeloid leukemia (AML) is a life-threatening malignancy, derived from hematopoietic stem and progenitor cells in the bone marrow. The present AML classification system separates two main groups according to the presence or absence of defined genetic abnormalities like specific fusion genes or gene mutations. Cases which lack these marker abnormalities are further subclassified according to their differentiation status [1].

Deregulated differentiation is a general feature of cancer and a major factor in leukemogenesis [2,3]. Normal hematopoiesis is mainly controlled at the transcriptional level [4,5,6]. Therefore, aberrant activity of developmental transcription factors (TFs) frequently underlies differentiation defects in leukemia. For example, the megakaryocyte-erythroid progenitor (MEP) physiologically differentiates into one of the two indicated lineages, controlled by, inter alia, Zn-finger TF GATA1, ETS-family TF FLI1, and homeodomain TF IRX1. Accordingly, the mutation or aberrant expression of GATA1, FLI1, or IRX-related genes contributes to the development of megakaryocytic or erythroid leukemia [7,8,9,10].

Homeodomains enable corresponding TFs to undergo sequence-specific DNA-binding and interaction with cofactors. They are encoded by conserved homeoboxes, which serve to arrange these TFs into classes and subclasses [11,12]. Homeobox genes generally regulate developmental processes in cells and tissues. Accordingly, eleven genes from the 48 member-strong NKL subclass are physiologically expressed in the course of hematopoiesis and have been summarized as the hematopoietic NKL-code [13]. Of note, 35 NKL homeobox genes are reportedly deregulated in hematopoietic malignancies, highlighting their oncogenic potential. In particular AML subtypes, aberrantly activated NKL homeobox genes include physiological members (such as NKX2-3) and subclass members which are normally silent in the hematopoietic compartment (such as NKX2-4) [13,14]. Thus, NKL homeobox genes play important roles in both normal and abnormal hematopoiesis.

Glucose and glycolytic enzymes are central players in cellular metabolism, generating energy and biosynthetic building blocks. In cancer, this highly orchestrated metabolic system is frequently disturbed [15]. Accordingly, the renowned Warburg effect describes increased aerobic glycolysis and lactate production in cancer cells including AML [15,16]. Furthermore, specific modifications of chromatin proteins represent important regulators of gene activities. These modifications strongly depend on the availability of particular metabolites [17]. Therefore, the knowledge of deregulated metabolic processes and their mechanistic principles may help reveal effective approaches for therapeutic interventions in cancer treatment [15,17].

Recently, Lopez-Pajares and coworkers reported that TF IRF6 binds glucose, promoting IRF6 dimerization and hence altering its DNA-binding specificity and target gene selection. This mechanism plays a basic role in keratinocyte differentiation [18]. Thus, IRF6 implements a novel mechanism using metabolites to regulate gene activities and differentiation processes. Recently, we reported that overexpressed IRF6, together with ETV2 and HEY1, aberrantly activates NKL homeobox gene NKX2-4 in erythroid AML cell line OCI-M2. NKX2-4 mutually activates ETV2 and HEY1, and inhibits the expression of master regulator FLI1, which normally supports differentiation into megakaryocytes while repressing differentiation into erythrocytes [14]. Here, we used this cell line model to investigate the role of glucose and IRF6 in the regulation of oncogene activities in AML.

## 2. Materials and Methods

### 2.1. Cell Lines and Treatments

AML cell lines OCI-M2, F-36P, TF-1 and THP-1 were used in this study. They are held by the DSMZ (Braunschweig, Germany) and cultivated as described on the website (www.dsmz.de, accessed on 30 March 2026). They had been authenticated and tested negative for mycoplasma infection by our in-house service. Cells were treated with 3.5 mg/mL glucose or 3.5 mg/mL 2-deoxy-glucose (2-DG), and 100 µM etoposide dissolved in DMSO, obtained from Sigma-Aldrich (Taufkirchen, Germany). Modification of selected gene expression levels was performed using gene specific siRNA oligonucleotides with reference to AllStars negative Control siRNA (siCTR) obtained from Qiagen (Hilden, Germany). Overexpression studies were performed using commercial cDNA-constructs for ETV2 and PFKL cloned into expression vector pCMV6 (Thermo Fisher Scientific, Darmstadt, Germany). Phosphorothioate-modified DNA oligonucleotides (PTOs) were designed to block IRF6 binding sites: PTO-11 (5′-GCCCTGAGAGTTTCGCTCAGGCTCAG-3′) for mono-IRF6 at IRF6, PTO-22 (5′-CTGAGTTTCACAGTGGATACTTCTTA-3′) for dimer-IRF6 at IRF6, and PTO-KLF32 (5′-AATGTTTCACAAACTCTTAGAAACTA-3′) for dimer-IRF6 at KLF3. PTO-41 (5′-TAGAAGCCCTAGCCAGGACTAGCACA-3′) was used as control, as described previously [19]. Modified oligonucleotides were obtained from Eurofins MWG, Ebersberg, Germany. SiRNAs (80 pmol), plasmid DNA (2 µg), and PTOs (200 pmol) were transfected into 1 × 10^6^ cells by electroporation using the EPI-2500 impulse generator (Fischer, Heidelberg, Germany) at 350 V for 10 ms. Electroporated cells were harvested after 20 h cultivation.

For functional analyses we used the IncuCyte S3 Live-Cell Imaging Analysis System (Sartorius, Göttingen, Germany). Detection of apoptotic cells was performed using the IncuCyte Caspase-3/7 Green Apoptosis Assay diluted at 1:2000 (Sartorius). The Cell-by-Cell software tool was used for data analysis (Sartorius, Incucyte 2022B Rev2). Live-cell imaging experiments were performed twice with fourfold parallel tests.

### 2.2. Polymerase Chain Reaction (PCR) Analyses

Real-time quantitative (RQ)-PCR analysis was used to quantify transcripts/RNA and genomic DNA. Total RNA was extracted from cultivated cell lines using TRIzol reagent (Invitrogen, Darmstadt, Germany). Subsequently, cDNA was synthesized using 1 µg RNA, random priming and Superscript II (Invitrogen). Genomic DNA was extracted from cell lines using the QIAamp DNA mini-kit (Qiagen). RQ-PCR analyses were performed using the 7500 Real-time System, commercial buffer and primer sets (Applied Biosystems/Life Technologies, Darmstadt, Germany), or designed oligonucleotides. For normalization of expression levels, we quantified the transcripts of TATA box binding protein (TBP). For normalization of DNA copy numbers, we used the locus of MEF2C. The following oligonucleotides were used (obtained from Eurofins MWG): MEF2C-1 5′-AGAAGGCTTATGAGCTGAGC-3′ and MEF2C-2 5′-AGACTGGCATCTCGAAGTTG-3′, GPI-1 5′-TTGCAGATCATCCTGGTGGGCCAGC-3′ and GPI-2 5′-CTGGTCTCCCTAGCGGGGAGCC-3′, PFKL-1 5′-TCTGGCCTGACATGTCCAGTGTGGC-3′ and PFKL-2 5-TGCACAGCCTTGGGGTGCTGGCTG-3′. Quantitative analyses were performed as biological triplicates, while PTO-transfections and genomic analyses were performed twice. RQ-PCR analyses were performed in triplicate. Standard deviations are presented in the figures as error bars. Statistical significance was assessed by the Mann–Whitney U-Test. The calculated *p*-values are indicated by asterisks (* *p* < 0.05; ** *p* < 0.01; *** *p* < 0.001; n.s. not significant).

### 2.3. Protein Analysis

Protein lysates from cell lines were prepared using SIGMAFast protease inhibitor cocktail (Sigma-Aldrich) for subsequent Western blot analysis. Proteins were transferred onto nitrocellulose membranes (Bio-Rad, Munich, Germany) via the semi-dry method and blocked with 5% dry milk powder dissolved in phosphate-buffered saline buffer (PBS). The following antibodies were used: alpha-Tubulin (Sigma, #T6199), IRF6 (OriGene Technologies, Wiesbaden, Germany, #UM500074), PFKL (Santa Cruz Biotechnology, Dallas, TX, USA, #sc-393713), and GPI (Santa Cruz Biotechnology, Dallas, TX, USA, #sc-271459). For loading, control blots were reversibly stained with Poinceau (Sigma-Aldrich), and detection of alpha-Tubulin (TUBA) was performed thereafter. Secondary antibodies were linked to peroxidase for detection by Western-Lightning-ECL (Perkin Elmer, Waltham, MA, USA). Documentation was performed using the digital system ChemoStar Imager (INTAS, Göttingen, Germany).

### 2.4. Bioinformatic Analyses

The reported RNAseq dataset LL-100 covers 100 leukemia/lymphoma cell lines [20]. Based on these data, the public tool DSMZCellDive (https://celldive.dsmz.de, accessed on 30 March 2026) illustrates gene expression levels for selected genes and cell lines as a bar chart or a heatmap [21]. The selection of genes encoding glycolytic enzymes was performed in correspondence to BRENDA pathways (www.brenda-enzymes.info, accessed on 30 March 2026) [22].

Consensus binding sites for TFs were obtained from the CIS-BP database (https://cisbp.ccbr.utoronto.ca, accessed on 30 March 2026) and used for the screening of genomic DNA sequences via the UCSC genome browser (https://genome.cse.ucsc.edu, accessed on 30 March 2026).

GPI and PFKL expressions were analyzed in 289 AML patients containing normal karyotypes or chromosomal rearrangements, using the public GEO dataset GSE61804 [23] and the associated online tool GEOR. For the overall survival curve analysis of AML patients, we used the public TCGA data from the Cancer Genome Atlas [24] and applied the tool Gene Expression Profiling Interactive Analysis (GEPIA, version 1.0) [25]. Gene expressions of GPI and PFKL in 108 AML patients were cut off by the median to 50% high and 50% low samples. The *p*-value for the Log-rank test and 95% confidence interval are indicated in the survival plot.

### 2.5. Genomic Profiling Analysis

For genomic profiling, the genomic DNA of cell line OCI-M2 was prepared using the QIAamp DNA mini-kit (Qiagen). Labelling, hybridization and scanning of Cytoscan HD arrays were performed by the Genome Analytics Facility located at the Helmholtz Centre for Infection Research (Braunschweig, Germany), according to the manufacturer’s protocols (Affymetrix, High Wycombe, UK). These arrays are based on single-nucleotide polymorphisms (SNPs) and allow the determination of copy number states. Data were interpreted using the Chromosome Analysis Suite software version 3.1.0.15 (Affymetrix, High Wycombe, UK), and copy number alterations were determined accordingly.

### 2.6. Metabolite Analysis

OCI-M2 cells (2 × 10^6^) were treated for siRNA-mediated knockdown of PFKL and harvested after 20 h. The treated cells were washed twice in PBS, and the pellets were frozen in liquid nitrogen and stored at −80 °C. Metabolites were extracted with 250 µL methanol spiked with 4% ribitol solution (0.2 mg/mL) as internal standard for 15 min in an ultrasound path. Subsequently 250 µL water and 250 µL dichloromethane were added. Following vigorous mixing, the samples were centrifuged for 4 min at 10,000× *g*. Then, 200 µL of the polar phase was dried under vacuum. Analysis of the metabolites glucose and lactate were performed by the gas chromatography–mass spectrometry approach with a two-step derivatization using methoxyamine hydrochloride solution (20 mg/mL in pyridine) and N-methyl-N-(trimethylsilyl)-trifluoracetamide. Samples were measured and analyzed as described previously [26]. Statistical significance between knockdown and control samples was assessed by the Mann–Whitney U-Test, and the calculated *p*-values are indicated by asterisks (** *p* < 0.01; *** *p* < 0.001).

## 3. Results

### 3.1. High Glucose Alters Oncogene Expression in AML Cell Line OCI-M2

**Figure 1:**
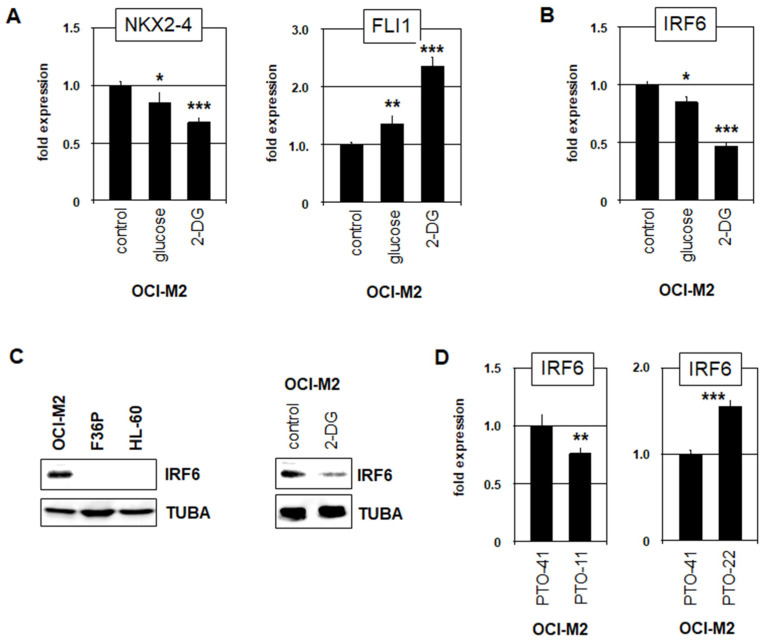
Glucose impacts expression of oncogenic TFs in AML. (**A**) RQ-PCR analysis of NKX2-4 (**left**) and FLI1 (**right**) in OCI-M2 after treatment with glucose or 2-deoxy-glucose (2-DG). (**B**) RQ-PCR analysis of IRF6 in OCI-M2 after treatment with glucose or 2DG. (**C**) Western blot analysis of IRF6 in selected AML cell lines (**left**) and in OCI-M2 after treatment with 2DG (**right**). TUBA served as loading control. (**D**) RQ-PCR analysis of IRF6 in OCI-M2 after treatment of enhancer-inhibition. PTO-11 targets the mono-IRF6 binding site, PTO-22 targets the dimer-IRF6 binding site, PTO-41 served as control. Quantitative analyses were performed in biological triplicates, PTO-treatments in duplicates, and RQ-PCR in triplicates. Statistical significance was assessed by Mann–Whitney U-Test, and the calculated *p*-values are indicated by asterisks (* *p* < 0.05; ** *p* < 0.01; *** *p* < 0.001).

Recently, we reported that IRF6 aberrantly activates the expression of NKX2-4, which in turn represses FLI1 in AML cell line OCI-M2 [14]. In keratinocytes, IRF6 interacts with glucose, which promotes dimerization, thus altering its DNA-binding specificity [18]. To analyze this regulatory mechanism in AML, we used cell line OCI-M2 as a model. Treatment of OCI-M2 with high glucose or its stable derivate 2-deoxy-glucose (2-DG) resulted in reduced activity of NKX2-4 and elevated expression of FLI1 (Figure 1A), indicating that IRF6 lost its capacity to activate NKX2-4 by glucose-mediated dimerization. However, this treatment also resulted in reduced expression of IRF6 itself as shown by RQ-PCR (Figure 1B) and Western blot analysis (Figure 1C), explaining the observed downregulation of its target gene NKX2-4.

IRF6 reportedly autoregulates its transcription, suggesting that this process was disturbed by glucose-mediated dimerization. Rahimov and colleagues identified an IRF6-binding site within an IRF6-enhancer which plays a role in cleft palate emergence when mutated [27]. Analysis of the regulatory upstream region of IRF6 demonstrated that this binding site only interacts with mono-IRF6, while we additionally identified another potential binding site which might interact with dimer-IRF6 (Figure S1A). Thus, their differing impact on IRF6 activity may underlie the observed glucose-mediated effect.

KLF3 is a reported IRF6 target gene in keratinocytes [18], which is also expressed in AML cell lines (Figure S1B), and serves as a prognostic marker in AML [28,29]. In its regulatory region, we confirmed the presence of a binding site for dimer-IRF6, while excluding those for mono-IRF6. Treatment of OCI-M2 with glucose/2-DG resulted in KLF3 activation (Figure S1B), reflecting the reported effect of glucose-mediated transition from mono-IRF6 to dimer-IRF6 in this cell line: dimerized IRF6 binds and activates KLF3 expression. Treatment of AML cell line TF-1 with high glucose significantly reduced expression of IRF6 and activated KLF3 (Figure S1C), supporting the results for OCI-M2.

To inhibit the autoregulatory IRF6 binding sites, we performed an enhancer-inhibition assay as described previously [19], using sequence-specific phosphorothioate-modified oligonucleotides in comparison to an unrelated control. Inhibition of the mono-IRF6 site resulted in reduced IRF6 expression while inhibition of the dimer-IRF6 site mediated IRF6 activation (Figure 1D). In contrast, inhibition of the dimer-IRF6 site at KLF3 resulted in reduced KLF3 expression, confirming its activating role at this locus (Figure S1B). Taken together, these results showed that mono-IRF6 autoactivated, while glucose-induced dimer-IRF6 autoinhibited IRF6 transcription and activated KLF3 expression. Thus, low glucose levels indirectly activate oncogene NKX2-4 via reduced IRF6 dimerization and the subsequent autoactivation of IRF6 expression.

### 3.2. Glycolytic Enzymes GPI and PFKL Are Aberrantly Overexpressed in OCI-M2

**Figure 2:**
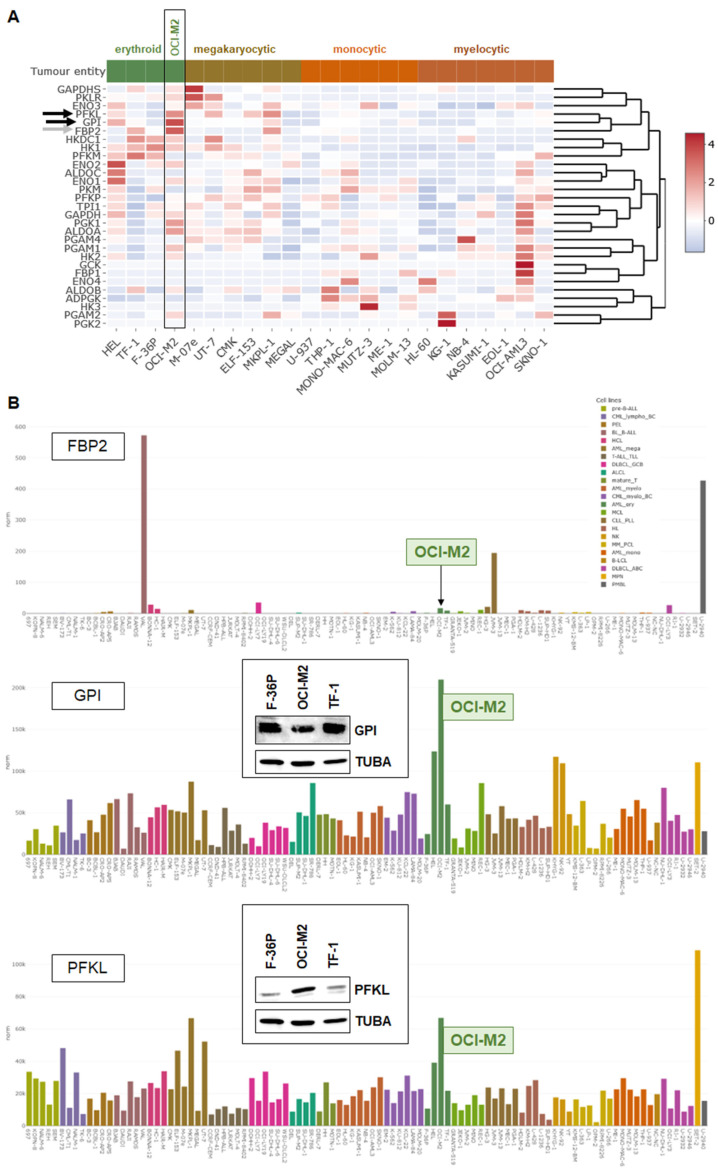
Overexpression of glycolytic genes in AML cell lines. (**A**) Heatmap showing expression levels of all glycolytic genes in erythroid, megakaryocytic, monocytic and myelocytic AML cell lines according to public RNA-seq data. Cell line OCI-M2 is boxed. (**B**) Bar charts showing expression levels of selected glycolytic genes, including FBP2, GPI and PFKL, according to public RNA-seq data from 100 leukemia/lymphoma cell lines. Color codes indicate the tumor entities. Erythroid AML cell line OCI-M2 is highlighted. Heatmap and bar charts were generated via the public DSMZCellDive tools. Western blot analyses of GPI and PFKL in selected AML cell lines are inserted. TUBA served as loading control.

To examine whether aberrantly expressed glycolytic enzymes are responsible for reduced glucose levels in OCI-M2, we screened their gene activities by analyzing our public RNA-seq data in 23 AML cell lines including OCI-M2 (Figure 2A). Overall, this exercise indicated elevated expression levels in erythroid and megakaryocytic AML cell lines. In particular, erythroid AML cell line OCI-M2 showed enhanced expression of FBP2, GPI and PFKL (Figure 2A). However, more detailed analyses of these three genes in 100 leukemia/lymphoma cell lines showed that the absolute FBP2 transcript levels were insignificantly low in AML cell lines, while confirming the conspicuously high expression levels of GPI and PFKL in OCI-M2 (Figure 2B). Elevated expression of PFKL was also demonstrated at the protein level via Western blot, while GPI showed rather reduced protein levels (Figure 2B). Thus, our data highlight enhanced expression of glycolytic member PFKL in OCI-M2.

**Figure 3:**
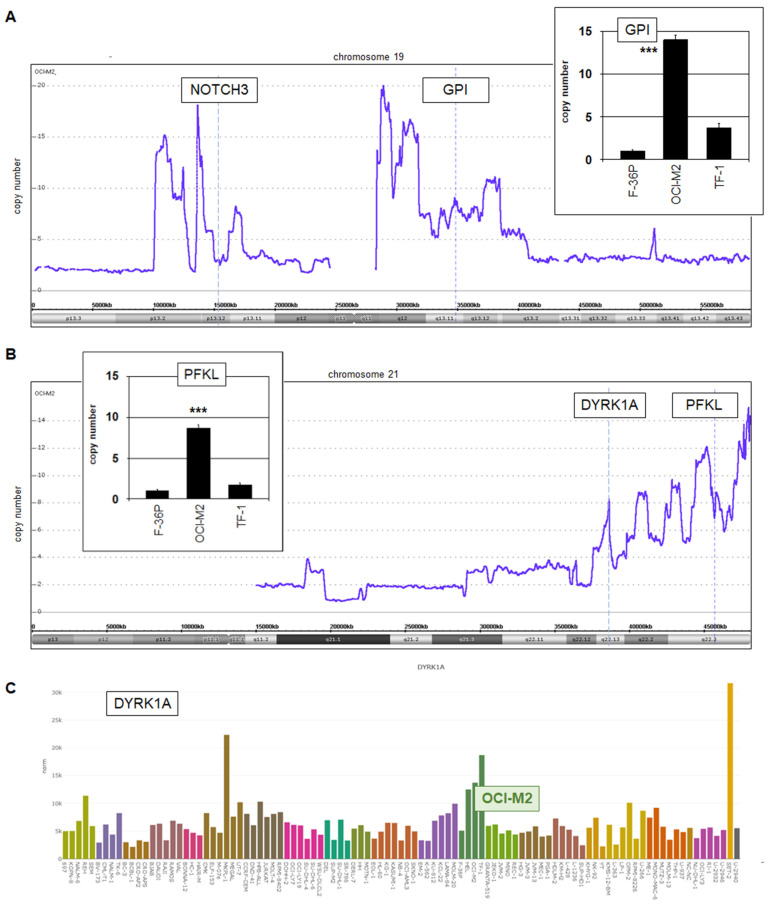
Genomic aberrations target GPI, NOTCH3, PFKL and DYRK1A. (**A**) Copy number analysis by genomic profiling shows complex focal amplifications of chromosome 19, targeting the genes NOTCH3 (19p13) and GPI (19q13). RQ-PCR analysis quantified the copy number of GPI in three AML cell lines (insert), confirming the profiling data for OCI-M2. (**B**) Copy number analysis by genomic profiling shows complex focal amplifications of chromosome 21q22, targeting the genes DYRK1A and PFKL. RQ-PCR analysis quantified the copy number of PFKL in three AML cell lines (insert), confirming the profiling data for OCI-M2. (**C**) Bar charts showing expression levels of DYRK1A according to public RNA-seq data from 100 leukemia/lymphoma cell lines. The color code indicates the tumor entities, as shown in Figure 2. Cell line OCI-M2 is highlighted. Quantitative analyses were performed in biological duplicates and RQ-PCR in triplicates. Statistical significance was assessed by Mann–Whitney U-Test and the calculated *p*-values are indicated by asterisks (*** *p* < 0.001).

The genes GPI and PFKL are located at the chromosomal positions 19q13.11 and 21q22.3, respectively. To see if their loci are aberrantly rearranged, we performed genomic profiling analysis using SNP-arrays. The data indicated that both genes are part of complex amplifications (Figure 3A,B), which may underlie their enhanced activities. We confirmed their raised copy numbers by RQ-PCR analysis, showing a more than 10-fold amplification of GPI and a nearly 10-fold amplification of PFKL (Figure 3A,B). Interestingly, the amplification pattern at chromosome 21q22 shows regularly staggered peaks, resembling those we previously described for chromothripsis in Hodgkin lymphoma cell line L-1236 at chromosomal regions 3q and 9q [30]. Moreover, the gene DYRK1A is located at 21q22.13, thus forming part of this rearranged region, and overexpressed in OCI-M2 (Figure 3B,C). DYRK1A is a reportedly highly expressed gene activated via chromothripsis in myeloproliferative neoplasms [31], thus supporting our interpretation that amplified DYRK1A and PFKL in AML cell line OCI-M2 are also aberrantly overexpressed by this type of chromoanagenesis.

### 3.3. Upstream Regulators and Downstream Targets of PFKL

**Figure 4:**
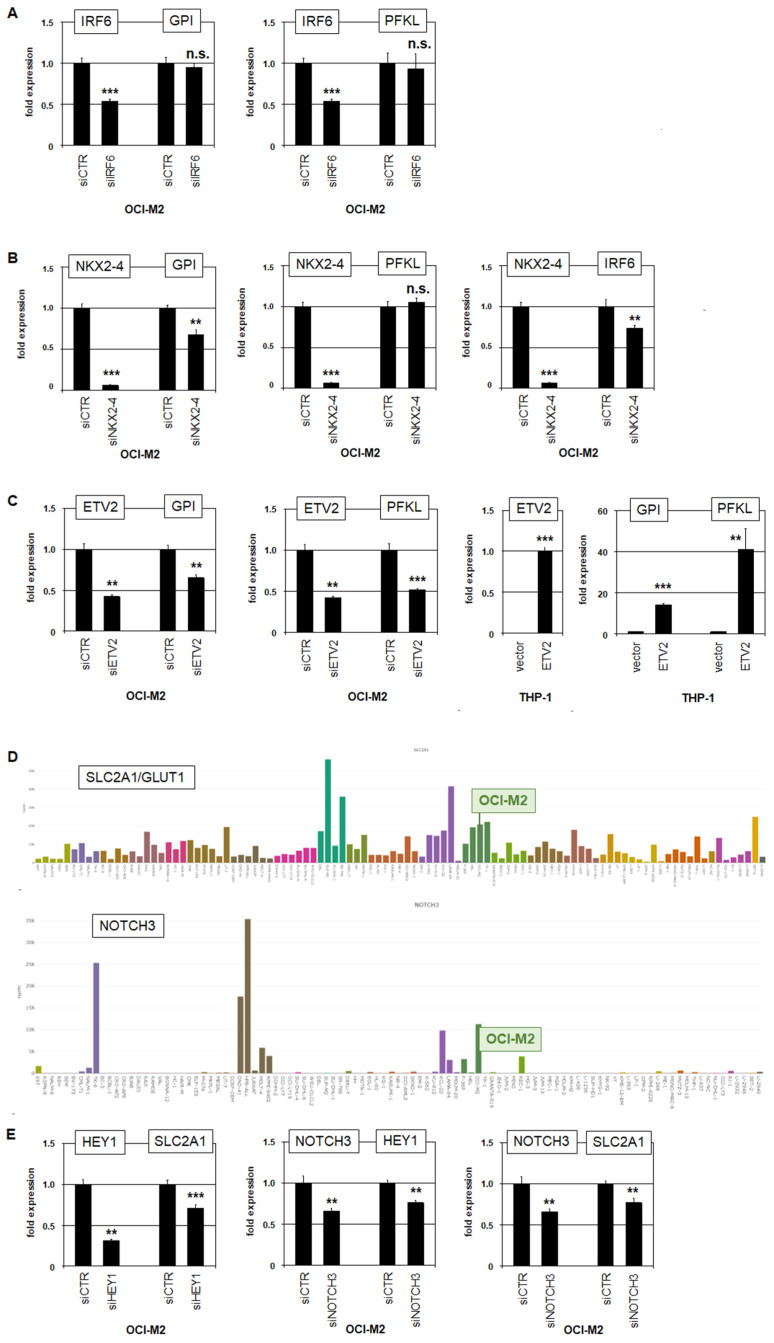
Oncogenic TFs activate genes of glucose metabolism. (**A**) RQ-PCR analysis of IRF6, GPI and PFKL in OCI-M2 after treatment for siRNA-mediated knockdown of IRF6. (**B**) RQ-PCR analysis of NKX2-4, GPI, PFKL and IRF6 in OCI-M2 after treatment for siRNA-mediated knockdown of NKX2-4. (**C**) RQ-PCR analysis of ETV2, GPI and PFKL in OCI-M2 after treatment for siRNA-mediated knockdown of ETV2 (**left**), and in THP-1 after forced expression of ETV2 (**right**). (**D**) Bar charts showing expression levels of SLC2A1 (**above**) and NOTCH3 (**below**) according to public RNA-seq data from 100 leukemia/lymphoma cell lines. Color codes indicate tumor entities, as shown in Figure 2. Cell line OCI-M2 is highlighted. (**E**) RQ-PCR analysis of HEY1, SLC2A1 and NOTCH3 in OCI-M2 after treatment for siRNA-mediated knockdown of HEY1 (**left**) and NOTCH3 (**middle** and **right**). Quantitative analyses were performed in biological triplicates and RQ-PCR in triplicates. Statistical significance was assessed by Mann–Whitney U-Test and calculated *p*-values are indicated by asterisks (** *p* < 0.01; *** *p* < 0.001; n.s. not significant).

GPI encodes the glycolytic enzyme glucose-6-phosphate isomerase which catalyzes the reversible transformation between glucose-6-phosphate and fructose-6-phosphate. PFKL encodes phosphofructokinase L which catalyzes the irreversible transformation of fructose-6-phosphate into fructose-1,6-bisphosphate, and represents a metabolic driver and major regulatory checkpoint for the whole glycolytic pathway [32]. To analyze whether members of the reported oncogenic network in OCI-M2 contribute to the aberrant overexpression of GPI and/or PFKL, we performed siRNA-mediated knockdown experiments. However, our results excluded any impact of IRF6 on their expression (Figure 4A). In contrast, NKX2-4 activated GPI as well as IRF6, but not PFKL (Figure 4B), and ETV2 activated both GPI and PFKL (Figure 4C). Activation of GPI and PFKL was also shown in AML cell line THP-1 after the forced expression of ETV2 (Figure 4C), supporting this regulatory impact.

Glucose-transporter SLC2A1 (alias GLUT1) has been reported as an activated target from NOTCH1-driven HES1 in colon cancer [33]. Therefore, we speculated whether the HES1-related factor HEY1 may similarly activate SLC2A1 in OCI-M2, which accordingly showed elevated transcript levels (Figure 4D). Knockdown experiments indeed demonstrated that HEY1 activated SLC2A1, and that aberrantly expressed NOTCH3 activated HEY1 (Figure 4D,E). Of note, NOTCH3 is located at chromosomal position 19p13 and is genomically duplicated, which may underlie its enhanced expression (Figure 3). Thus, all reported oncogenic TFs in AML cell line OCI-M2 play a role in the deregulation of glucose metabolism.

**Figure 5:**
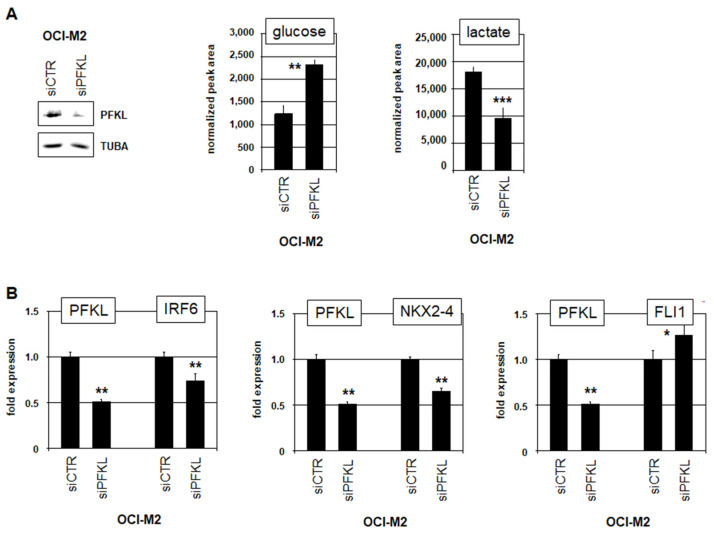
Overexpressed PFKL reduced glucose levels and activated oncogenes in AML. (**A**) Western blot analysis of PFKL in OCI-M2 treated for siRNA-mediated knockdown of PFKL. TUBA served as loading control (**left**). Gas chromatography–mass spectrometry quantification of intra-cellular glucose (**middle**) and lactate (**right**) in OCI-M2 treated for siRNA-mediated knockdown of PFKL. (**B**) RQ-PCR analysis of PFKL, IRF6, NKX2-4 and FLI1 in OCI-M2 after treatment for siRNA-mediated knockdown of PFKL. Quantitative analyses were performed in biological triplicates and RQ-PCR in triplicates. Statistical significance was assessed by Mann–Whitney U-Test and the calculated *p*-values are indicated by asterisks (* *p* < 0.05; ** *p* < 0.01; *** *p* < 0.001).

Expression analysis of GPI and PFKL in 289 AML patients containing either normal or rearranged karyotypes was performed using the public GEO dataset GSE61804 [23]. The data demonstrated aberrant overexpression of both genes in subsets of both patient groups (Figure S2), indicating an oncogenic role for GPI and PFKL irrespective of the karyotype. However, analysis of 106 AML patients from the TCGA dataset showed that elevated PFKL expression is associated with reduced survival, contrasting GPI (Figure S3). Consistently, PFKL represents an important checkpoint for the regulation of glycolytic activity and is frequently overexpressed in cancer [34]. Therefore, our final investigations focused on the particular role of PFKL in AML cell line OCI-M2. SiRNA-mediated knockdown of PFKL was confirmed by Western blot and resulted in elevated glucose levels and reduced lactate levels (Figure 5A), supporting its central role in the regulation of glycolysis. Furthermore, PFKL knockdown resulted in reduced expression of IRF6 and NKX2-4, and the elevated expression of FLI1 (Figure 5B), while forced expression of PFKL reduced KLF3 transcription in NKX2-4 negative AML cell line TF-1 (Figure S1C). Collectively, our data showed that aberrantly overexpressed PFKL reduced the intra-cellular glucose level, which in turn may support the availability of mono-IRF6 at the expense of glucose-mediated dimer-IRF6, and the autoactivation of IRF6. Finally, mono-IRF6 activates NKX2-4, which suppresses the inhibitor of erythropoiesis FLI1 and disturbs corresponding differentiation processes in OCI-M2.

**Figure 6:**
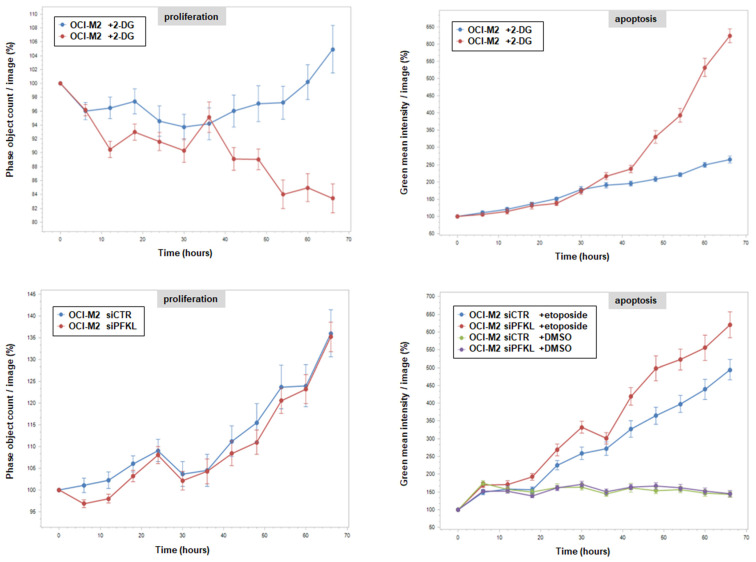
Functional effects of high glucose and PFKL knockdown. Live-cell imaging analysis of AML cell line OCI-M2 after treatment with 2-DG (**above**) and siRNA-mediated knockdown of PFKL (**below**). Of note, apoptosis was additionally induced by treatment with etoposide.

To analyze additional effects of 2-DG and enhanced PFKL in AML, we performed live-cell imaging of OCI-M2 cells treated with 2-DG or siRNA-mediated knockdown of PFKL for 70 h (Figure 6). The data indicated that 2-DG treatment inhibited proliferation and induced apoptosis. In contrast, PFKL knockdown showed no impact on proliferation but enhanced etoposide-induced apoptosis. Taken together, our data indicated that PFKL overexpression deregulated myeloid differentiation and supported survival in model cell line OCI-M2, highlighting its oncogenic role in AML. 2-DG supported IRF6-dimerization, inhibited proliferation and induced apoptosis, thus representing a potent drug to treat this type of AML.

## 4. Discussion

**Figure 7:**
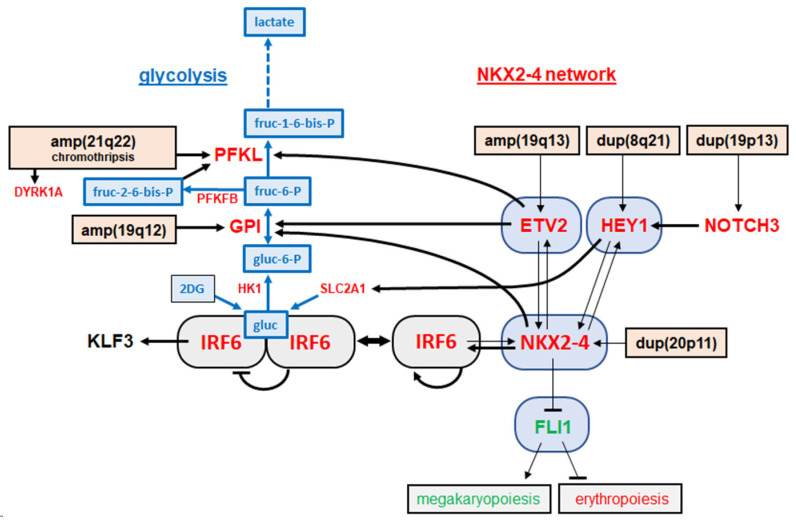
Expanded oncogenic network in AML cell line OCI-M2. This illustration summarizes the results of this study, showing the glycolytic pathway on the left and the previously reported oncogenic NKX2-4 network on the right [14]. Highly expressed genes are indicated in red, intermediately in black, and downregulated genes in green. Sugars are indicated in blue. Genomic aberrations are black-boxed. Gene regulatory impacts investigated in this study are indicated by fat arrows and published impacts by thin arrows and lines.

In this study, we expanded our previously reported oncogenic network which aberrantly activates NKL homeobox gene NKX2-4 in an erythroid AML model [14]. We uncovered a regulatory role for glucose on the expression of activator IRF6 inferred by the reported mechanism of glucose-mediated dimerization of this TF [18]. The data indicated that aberrantly overexpressed glycolytic PFKL reduced glucose levels, which activated NKX2-4 expression via autoactivated mono-IRF6. Figure 7 illustrates and summarizes our results, which combine deregulated glycolysis with aberrant TF activities and are further discussed below.

Deregulated metabolism plays a general role in carcinogenesis, while different pathways, enzymes and metabolites may be affected [15]. In AML, the pentose phosphate pathway and glycolysis are reportedly disturbed, contributing to leukemogenesis and chemoresistance [35,36]. Furthermore, these pathways are responsible for elevated lactate levels, which remodel the tumor microenvironment and activate tumor cell proliferation, thus representing a risk factor for this malignancy [37,38]. In erythroid AML cell line HEL, KDM1A stabilizes the erythropoietic TF GATA1, which activates the expression of glucose-transporter SLC2A1 and selected glycolytic enzymes [39], highlighting the role of deregulated glycolysis in this type of AML.

In erythroid AML cell line OCI-M2, we detected aberrant overexpression of the glycolytic genes GPI and PFKL, while only PFKL was also overexpressed at the protein level. NKL homeodomain TF NKX2-4 activated GPI transcription, while ETS-family TF ETV2 activated both GPI and PFKL. This is the first report implicating NKX2-4 in the regulation of any glycolytic enzyme. In contrast, ETV2 reportedly activates glycolysis in bone regeneration [40], thus supporting our results in the context of leukemia. In neuroblastoma, IRF6 indirectly impacts glycolysis via inhibition of phosphoglucomutase PGM1 [41]. Of note, this enzyme connects the glycolytic pathway with the storage of glucose in the form of glycogen. Accordingly, PGM1 is reduced in hepatocellular carcinoma, shifting glucose into glycolysis [42]. Interestingly, OCI-M2 also exhibited PGM1 downregulation (Figure S4), indicating that IRF6 may also suppress PGM1 and support glycolysis in AML. Finally, we showed that basic helix–loop–helix TF HEY1 activated glucose-transporter SLC2A1, resembling its regulation by HES1 in colon cancer [33]. In pancreas cancer, basic helix–loop–helix TF TWIST1 aberrantly activates SLC2A1 in addition to the glycolytic enzymes HK2, ENO1 and PKM2 [43], demonstrating that developmental TFs of this type also perform deregulation of glucose metabolism.

Our study highlights the oncogenic role of overexpressed PFKL in erythroid AML. PFKL represents a main checkpoint for glycolysis. Consistent with our observations, PFKL overexpression has been described in several cancers [34]. We showed that the locus of PFKL is amplified in OCI-M2. Furthermore, the striking pattern of this amplicon at 21q22 resembles that described for chromothripsis in Hodgkin lymphoma cell line L-1236 [30] and may be referred to as a chromothripsis-like pattern. Chromothripsis has been reported in both lymphoid and myeloid malignancies, including myelodysplastic syndromes and AML [30,31,44,45]. Chromothripsis is a class of catastrophic chromosome instability or chromoanagenesis, and may affect the focal amplification of the rearranged genomic segments [46], as observed in OCI-M2 and L-1236. However, additional analyses are required to confirm this conclusion. In addition, fructose-2-6-bis-phosphate operates as allosteric activator of PFKL and is generated by the enzyme PFKFB [47]. Interestingly, OCI-M2 showed enhanced expression of all four PFKFB isogenes, namely PFKFB1, PFKFB2, PFKFB3 and PFKFB4 (Figure S4), indicating that this mechanism of PFKL activation may also play a functional role in this AML cell line.

Functional analyses in OCI-M2 indicated that PFKL inhibits apoptosis. These results correspond to our findings that AML patients expressing elevated PFKL show reduced overall survival. Treatment of OCI-M2 with 2-DG altered myeloid differentiation via FLI1 expression, induced apoptosis, and inhibited proliferation. This drug imitates glucose in IRF6 regulation but inhibits glycolysis, thus representing a suitable treatment option for corresponding AML patient subsets.

PFKL overexpression resulted in reduced glucose levels in OCI-M2, which impacted the expression of IRF6. IRF6 is able to interact with DNA as a monomer or dimer [48]. Furthermore, IRF6 may activate or repress its target genes, implying a complex mode of regulation. Lopez-Pajares and coworkers reported a novel mechanism of glucose-mediated dimerization of IRF6, which alters the selection of DNA-binding and thus its target genes [18]. Our data indicated that mono-IRF6 autoactivated IRF6 via a reported binding site, which plays a role in cleft palate generation [27]. In contrast, dimer-IRF6 activated KLF3 but inhibited IRF6 via a different binding site and concomitantly reduced levels of mono-IRF6. Thus, in OCI-M2, glucose operates as an IRF6-regulator, which alters both potential and activity of this TF.

NKL homeobox gene NKX2-4 and glucose-regulated IRF6 are mutual activators and central to an oncogenic network which deregulates myeloid differentiation. The perturbation of developmental TFs and their orchestrated differentiation processes is a common theme in AML. Model cell line OCI-M2 may have originated from MEPs which sustained a developmental arrest and thus generated erythroblastic AML via NKX2-4 mediated suppression of FLI1 [14]. IRF6 physiologically regulates adult erythropoiesis via interaction with master TFs GATA1 and TAL1, and the immune response in neutrophils and macrophages [49,50], indicating that altered glucose levels may also impact these processes. Of note, IRF6 target gene KLF3 may also play a role in the leukemogenesis of OCI-M2, because low KLF3 levels have been reported as a favorable prognostic marker in AML [28,29]. We showed that elevated glucose levels reduced the expression of oncogene NKX2-4 and simultaneously increased KLF3 expression. Thus, the forced elevation of glucose levels induced antagonistic effects when targeting glycolysis in this AML model. These data highlight unwanted side effects of pharmacological therapies, requiring special attention.

## 5. Conclusions

This study illuminates the oncogenic impact of reduced glucose levels in AML. Aberrant activation of glycolytic PFKL and the direct regulatory connection between glucose and TF IRF6 drive an oncogenic network which deregulates myeloid differentiation and supports survival. The provision of both a novel oncogenesis paradigm and a model cell line for its investigation may pave the way for novel therapeutic approaches to treat glucose-sensitive AML in the clinic.

## Data Availability

The original data presented in the study are openly available as indicated in the text.

## Supplementary Materials

The following supporting information can be downloaded at: https://www.mdpi.com/article/10.3390/cells15060569/s1, Figure S1: IRF6 binding site analysis at IRF6 and KLF3; Figure S2: Expression of GPI and PFKL in AML patients; Figure S3: Survival analysis of AML patients overexpressing GPI and PFKL; Figure S4: Expression of PGM1 and PFKFB in AML cell lines.

## Abbreviations

The following abbreviations are used in this manuscript:

2DG	2-deoxy-glucose
AML	Acute myeloid leukemia
TF	Transcription factor
