# Supplementary material for: Glycolytic Enzymes Are Part of an Oncogenic Network in AML
Source: Cells. 2026 Mar 23;15(6):569. doi: 10.3390/cells15060569 (PMC13024823; doi:10.3390/cells15060569)
Supplement: Supplementary file 1 [file cells-15-00569-s001.zip › cells-4180934-supplementary.pdf]

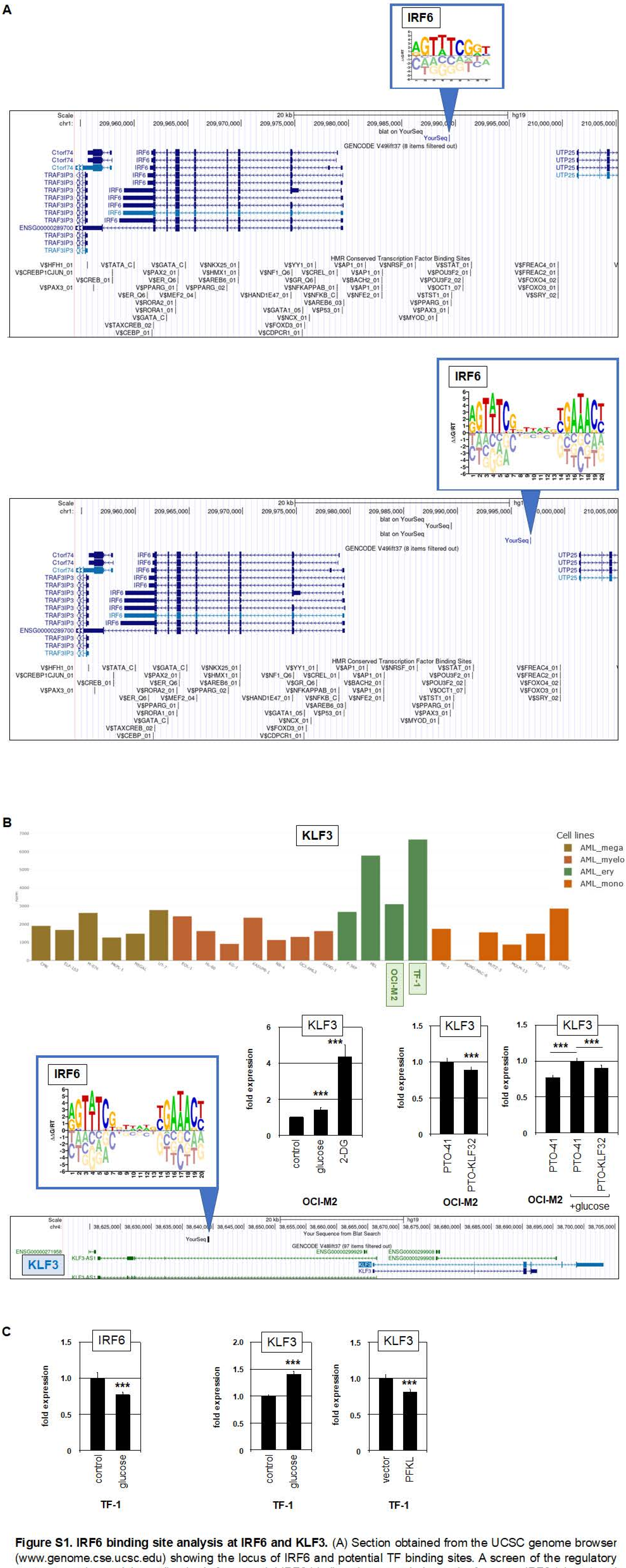

Figure S1. IRF6 binding site analysis at IRF6 and KLF3. (A) Section obtained from the UCSC genome browser (www.genome.cse.ucsc.edu) showing the locus of IRF6 and potential TF binding sites. A screen of the regulatory upstream region and the coding body for potential IRF6-binding sites revealed one site for mono-IRF6 (above) and another for dimer-IRF6 (below). The consensus sequences were obtained from the CIS-BP database (www.cisbp.ccbr.utoronto.ca). (B) Bar chart showing expression levels of KLF3 in AML cell lines according to RNA-seq data from the public dataset LL-100 (above). RQ-PCR analysis of IRF6 in OCI-M2 after treatment with glucose or 2-DG (left), of KLF3 in OCI-M2 after inhibition of dimer-IRF6 site (middle), and with concurrent addition of glucose (right). Section obtained from the UCSC genome browser showing the locus of IRF6 and the identified potential IRF6 binding site, representing a site for dimer-IRF6 (below). (C) RQ-PCR analysis of IRF6 (left) and KLF3 (middle) in TF-1 after treatment with glucose, and of KLF3 after forced expression of PFKL in TF-1 (right). Quantitative analyses were performed in biological triplicates, PTO-treatments in duplicates, RQ-PCR in triplicates. Statistical significance was assessed by Mann-Whitney U-Test and the calculated p-values were indicated by asterisks (*** $p<0.001$).

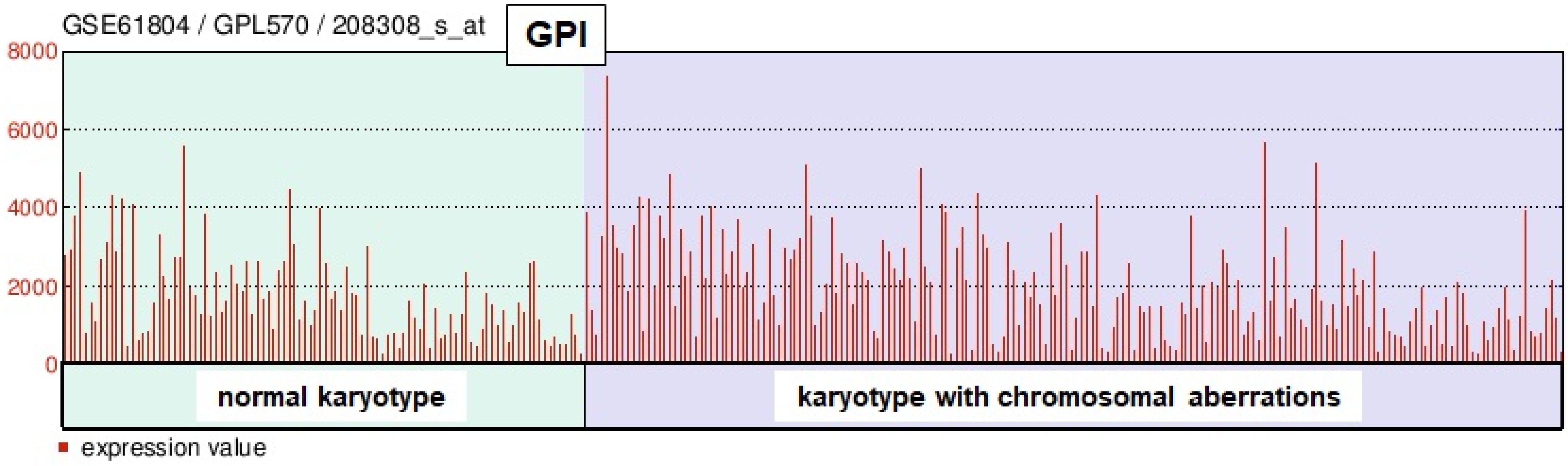

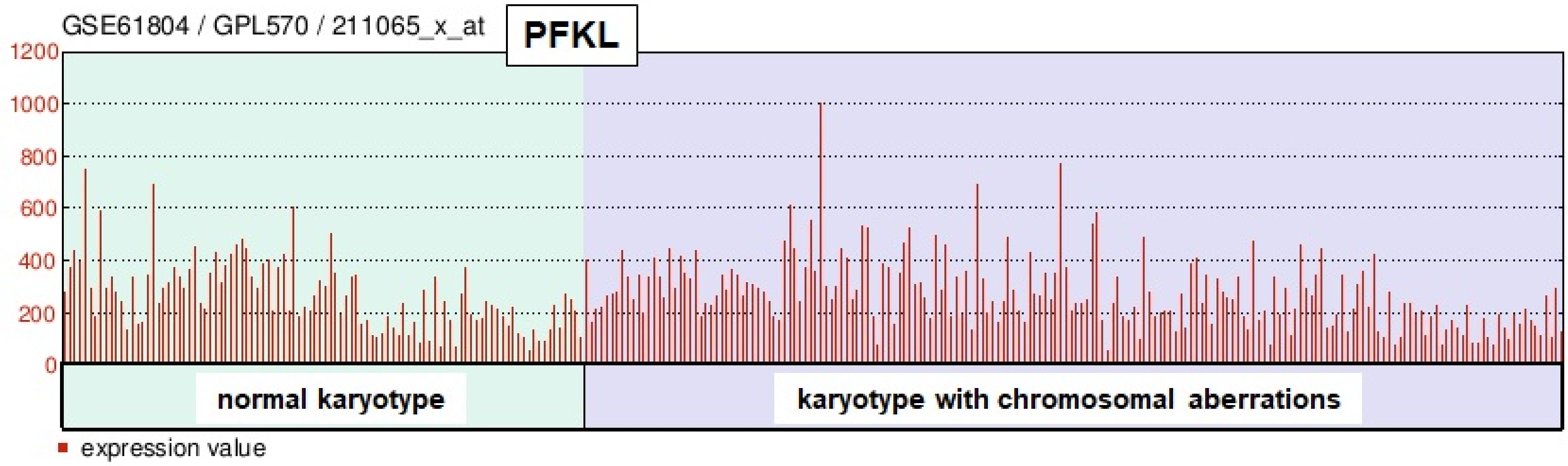

Figure S2. Expression analysis of GPI (above) and PFKL (below) in AML patients containing either normal karyotypes or chromosomal rearrangements, using GEO dataset GSE61804.

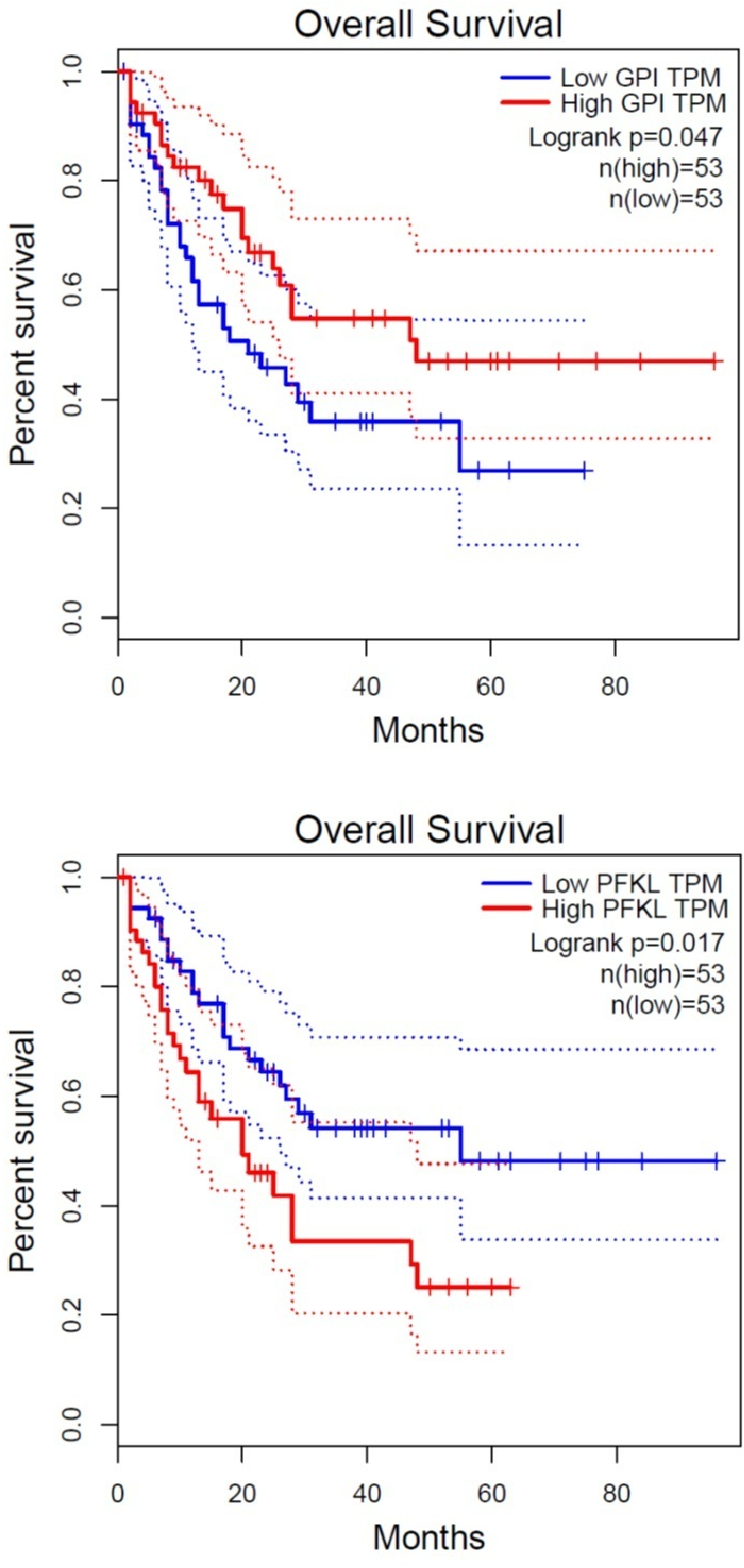

Figure S3. Survival analysis of AML patients expressing elevated GPI (above) and PFKL (below), using TCGA datasets.

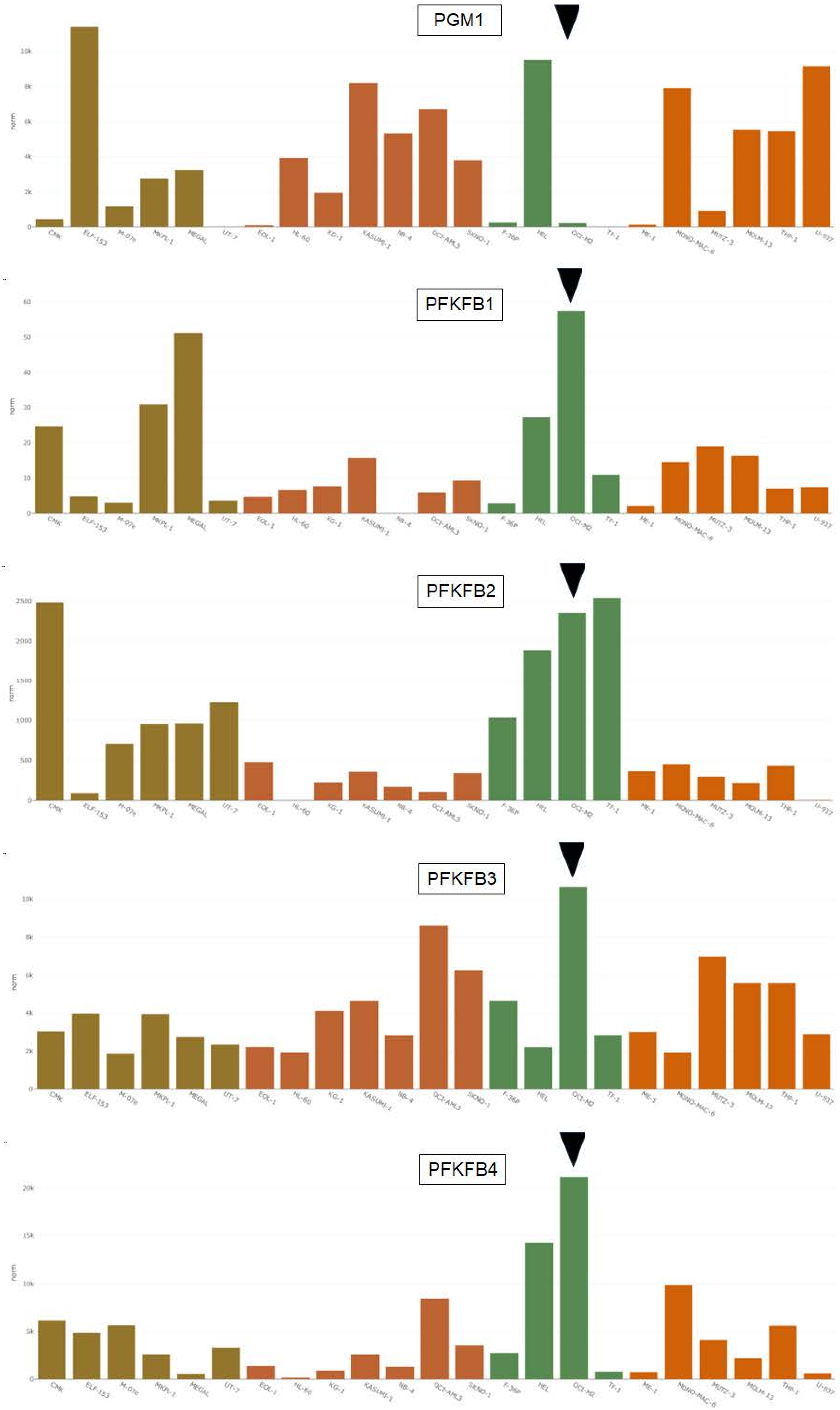

Figure S4. Expression of PGM1 and PFKFB in AML cell lines. Bar charts showing expression levels of PGM1, PFKFB1, PFKFB2, PFKFB3 and PFKFB4 in AML cell lines according to RNA-seq data from the public dataset LL-100. Cell line OCI-M2 is highlighted by an arrow head.
